# R409K mutation prevents acid-induced aggregation of human IgG4

**DOI:** 10.1371/journal.pone.0229027

**Published:** 2020-03-17

**Authors:** Hiroshi Namisaki, Seiji Saito, Keiko Hiraishi, Tomoko Haba, Yoshitaka Tanaka, Hideaki Yoshida, Shigeru Iida, Nobuaki Takahashi

**Affiliations:** 1 Alliance Development Group, Open Innovation Department R&D Division, Kyowa Kirin Co., Ltd., Tokyo, Japan; 2 Division of Pharmaceutical Cell Biology, Graduate School of Pharmaceutical Sciences, Kyushu University, Maidashi, Fukuoka, Japan; 3 Tokyo Research Park, Kyowa Kirin Co., Ltd., Tokyo, Japan; 4 Research Functions Unit, R&D Division, Kyowa Kirin Co., Ltd., Tokyo, Japan; University of Colorado Anschutz Medical Campus, UNITED STATES

## Abstract

Human immunoglobulin G isotype 4 (IgG4) antibodies are suitable for use in either the antagonist or agonist format because their low effector functions prevent target cytotoxicity or unwanted cytokine secretion. However, while manufacturing therapeutic antibodies, they are exposed to low pH during purification, and IgG4 is more susceptible to low-pH-induced aggregation than IgG1. Therefore, we investigated the underlying mechanisms of IgG4 aggregation at low pH and engineered an IgG4 with enhanced stability. By swapping the constant regions of IgG1 and IgG4, we determined that the constant heavy chain (CH3) domain is critical for aggregate formation, but a core-hinge-stabilizing S228P mutation in IgG4 is insufficient for preventing aggregation. To identify the aggregation-prone amino acid, we substituted the CH3 domain of IgG4 with that of IgG1, changing IgG4 Arg409 to a Lys, thereby preventing the aggregation of the IgG4 variant as effectively as in IgG1. A stabilizing effect was also recorded with other variable-region variants. Analysis of thermal stability using differential scanning calorimetry revealed that the R409K substitution increased the *T*m value of CH3, suggesting that the R409K mutation contributed to the structural strengthening of the CH3-CH3 interaction. The R409K mutation did not influence the binding to antigens/human Fcγ receptors; whereas, the concurrent S228P and R409K mutations in IgG4 suppressed Fab-arm exchange drastically and as effectively as in IgG1, in both *in vitro* and *in vivo* in mice models. Our findings suggest that the IgG4 R409K variant represents a potential therapeutic IgG for use in low-effector-activity format that exhibits increased stability.

## Introduction

Immunoglobulins are glycoproteins that recognize antigens and participate in biological defense as a result of the activation of their effector functions, involving complement-dependent cytotoxicity (CDC), phagocytosis, and antibody-dependent cellular cytotoxicity (ADCC). Immunoglobulins are classified into five classes: IgA, IgD, IgE, IgG, and IgM, and IgG antibodies. IgG antibodies are heterodimers composed of two heavy chains and two light chains and are further classified into four subclasses according to the constant region of their heavy chains: IgG1, IgG2, IgG3, and IgG4. To date, >50 monoclonal antibodies have been approved as drugs against cancers, chronic diseases, and autoimmune diseases, and >500 monoclonal antibody clinical trials are ongoing [1]. In 2018, 12 new therapeutic antibodies were approved for the treatment of a variety of diseases in the EU or USA [2]. These approved antibodies all belong to the subclass of IgG1, IgG2, or IgG4. Therapeutic IgG antibodies are selected according to their subclasses based on the desired functional action [3]. For example, therapeutic IgG1 antibodies are required for depleting target cells through IgG1 effector functions such as ADCC and CDC. Therefore, to increase the efficacy of these antibodies, technologies that enhance ADCC and CDC have been reported [4, 5]. Conversely, IgG2 and IgG4 antibodies are suitable for use either in the antagonist or agonist format because they present low cytotoxic potentials relative to IgG1 and IgG3 [6]. Most therapeutic antibodies introduced thus far are derived from the IgG1 subclass, although the IgG4 subclass has also been adapted to develop products that are to date on EU and US markets, such as natalizumab, pembrolizumab, ixekizumab, reslizumab, nivolumab, emicizumab, and galcanezumab [2]. Recently, there is an increase in the number of IgG4 formats in clinical development for application in cancer immunotherapies, such as in the case of target CD4–positive T cells. This is because of the low effector functions toward normal cells expressing as target cells [7]. Although ADCC activity was observed with the use of IgG1-type nivolumab, the antibody did not mediate ADCC [8].

IgG4 antibodies possess unique physicochemical and physiological properties. In humans, they can form half-antibodies comprising a single light chain and a single heavy chain, and these half-antibodies feature the intra-chain disulfide bonds without the inter-heavy chain disulfide bonds in the hinge domain. Currently, therapeutic IgG4 antibodies, such as nivolumab, are adapted from a human IgG4 variant containing a substitution, S228P, to prevent formation of half-antibodies [8, 9]. IgG4 antibodies are recognized as dynamic molecules that exhibit Fab-arm exchange *in vitro* and *in vivo*, i.e., the antibodies exchange Fab arms by swapping half-antibodies from another IgG4 molecule, which generates bispecific antibodies [10, 11]. Among the human IgG subclasses, Fab-arm exchange is only observed in IgG4, which is known to form a half-antibody. IgG4 Fab-arm exchange is suggested to provide a basis for the anti-inflammatory activity of IgG4 antibodies. [10]. IgG4 autoantibodies against muscle-specific kinase undergo Fab-arm exchange in patients with myasthenia gravis. [12]. The recombination of IgG4 therapeutics with endogenous IgG4 may affect pharmacokinetics and pharmacodynamics. Notably, the S228P mutation markedly inhibited Fab-arm exchange in *in vitro* and *in vivo* studies and human clinical trials [11, 13]. Other mutations that enhance constant heavy (CH) 3 interactions in IgG4 have also been reported to prevent Fab-arm exchange [14, 15].

Another feature of IgG4 antibodies is their increased susceptibility to aggregation at low pH as compared with IgG1 antibodies [16]. The aggregation of therapeutic antibodies can influence both the efficacy and safety of therapeutic treatments, such as immunogenicity, infusion reaction, and complement activation [17–19]. Currently, in biopharmaceutical industrial manufacturing, therapeutic antibodies are exposed to approximately pH 3.5 for 1 h (for elution) during Protein A-based purification followed by virus inactivation [20]. Therefore, antibody aggregates must be removed to the extent possible before the clinical application of the therapeutic antibodies. In the manufacturing process, optimal antibody molecules are those that are stabilized to prevent aggregate formation. To date, several studies have reported the aggregation of IgG4 antibodies at low pH, and even the hinge-stabilized S228P IgG4 mutant has been found to be as susceptible to aggregation as wild-type IgG4 at pH 4.0 [21, 22]. Furthermore, nivolumab harboring the S228P mutation in IgG4 was also reported to show 30% aggregation at pH 3.5 [23].

Although the S228P IgG4 mutant, as noted above, showed drastically suppressed Fab-arm exchange [11, 13], hinge stability alone was suggested to be inadequate for influencing acid-induced aggregation, and thus other sites were suggested to affect IgG4 aggregation. In contrast, IgG1 is stable and resistant to aggregation at low pH. The stability of IgG subclasses harboring the same variable regions was ranked thus: IgG1 > IgG2 > IgG4 [21, 22, 24]. Therefore, we identified the amino acid that causes human IgG aggregation by swapping the constant regions of IgG1 and IgG4. No previous study has comprehensively analyzed the specific amino acid residues in human IgG4 responsible for acid-induced aggregation. We also used amino acid substitutions to establish a stable IgG4 variant without altering the unique biological characteristics of human IgG4-format antibodies used in therapeutics.

## Materials and methods

### Cell lines

Raji and Ramos (human B lymphoma) cells were obtained from the American Type Culture Collection. Both cell lines were cultured in RPMI-1640 medium (Sigma-Aldrich) supplemented with 10% heat-inactivated FCS (Gibco). HEK-293F cells (Invitrogen) were cultured in freestyle medium (Invitrogen).

### Construction, expression, and purification of antibodies

Expression vectors for anti-CD20 antibody were constructed by cloning the VH and VL coding regions of C2B8 (GenBank accession numbers: VH: AR000013; VL: AR015962) in the expression vector N5KG1 (Biogen IDEC, Cambridge, MA, USA). The obtained constructs were transfected into HEK-293F cells, and antibodies were purified from the supernatants by using Protein A-conjugated Sepharose columns (GE Healthcare, Malmo, Sweden). We also generated antibody expression vectors encoding domain subclass-switched/swapped and/or substituted derivatives: IgG4, IgG4PE, IgG1144E, IgG4411P, IgG4414P, IgG4441PE, IgG4PE_Gln355Arg, IgG4PE_Glu356Asp, IgG4PE_Met358Leu, IgG4PE_Arg409Lys, IgG4PE_Glu419Gln, IgG4PE_Leu445Pro, and IgG4PE_Lys370Glu. Each numeral indicates EU numbering. IgG4PE is a human IgG4 variant harboring S228P and L235E mutations to prevent half-antibody formation and to reduce ADCC [6]. IgG1144E is an IgG1/IgG4PE hybrid antibody that contains the CH1-hinge region of IgG1 and the CH2-CH3 regions of IgG4PE, whereas IgG4411P is a hybrid whose CH1-hinge and CH2-CH3 regions are from IgG4PE and IgG1, respectively. The hybrid IgG4414P features CH1-hinge and CH3 regions from IgG4PE and CH2 from IgG1, and IgG4441PE is a hybrid containing CH1-hinge-CH2 regions from IgG4PE and CH3 from IgG1. IgG4PE_Gln355Arg, a variant of IgG4PE, harbors a Gln-to-Arg substitution at position 355 in the CH3 domain. The other IgG4PE variants mentioned also harbor single mutations in the CH3 domain, as noted in their names.

Antibodies purified using Protein A-conjugated Sepharose columns were buffer-exchanged into D-PBS (Gibco) using a desalting column (NAP25 column; GE Healthcare). Finally, antibody concentrations were adjusted to 1.0 mg/mL, and the purity of prepared samples was analyzed using SDS–PAGE.

### Low-pH-stress assay

Antibodies were incubated in 0.1 mM citrate buffer (pH 2.7) at pH 3.4 for 10 min and 60 min at 37°C using a PCR Thermocycler (Applied Systems, Foster City, CA, USA). The amount of acid solution for pH 3.4 was determined in separate preliminary experiments. Each antibody concentration was 0.2 mg/mL. For neutralization, the samples were treated with 500 mM phosphate buffer (pH 8.0) at 4°C. Experiments were performed in triplicate.

### Size-exclusion chromatography (SEC)

The amounts of small aggregates or degradants formed were analyzed by performing SEC-HPLC (LC-7A, Shimadzu Corp.) with a G3000SWXL column (i.d., 7.8 mm; length, 30 cm; Tosoh Corp.), as described previously [21]. The mobile phase contained 20 mM sodium phosphate (pH 7.0) and 500 mM NaCl. The experimental conditions were as follows: flow rate, 0.5 mL/min; detection wavelength, 215 nm; and analysis time, 30 min. The antibody protein monomer was eluted at approximately 15.9 min. The soluble aggregates were eluted earlier than the degraded species. Peaks of antibody protein monomers were identified by comparing the elution positions derived from the antibody solutions and that of a molecular weight marker (Cat No. 40403701; Oriental Yeast Co., Ltd.) used for gel-filtration HPLC. The aggregate or degradant contents were measured based on the corresponding peak areas using LabSolutions software.

### Differential scanning calorimetry (DSC)

The thermal stability of single domains was evaluated using DSC. Measurements were performed on 0.5 mg/mL IgG in acetate buffer (pH 5.0) or PBS buffer (pH 6.0/7.4) using a Micro Cal VP-Capillary DSC system (Malvern Instruments Ltd.). Temperature scans were performed from 25 to 100°C at a scan rate of 1°C/min. A buffer–buffer reference scan was subtracted from each sample scan before concentration normalization. Baselines were created in Origin 7.0 (OriginLab) through a cubic interpolation of the pre- and post-transition baselines.

### Antibody binding to cell-surface antigens

Cell-surface antigen binding was analyzed using flow cytometry. Raji cells (5 × 10^5^) were incubated with various concentrations of anti-CD20 antibody or anti-DNP human IgG4 control antibody (DNP_IgG4) for 50 min at 4°C. After washing, anti-CD20 antibodies bound on the cell surface were detected using goat anti-human IgG(H+L)-Alexa Fluor 488 (Invitrogen) and a FACSCanto II system (Beckman Coulter).

### Preparation of hexa-His-tagged recombinant soluble human Fc receptors (shFcγRs)

Recombinant shFcγRI, shFcγRIIa-131H, shFcγRIIb, shFcγRIIIa-158V, shFcγRIIIa-158F, and shFcγRIIIb were prepared as described previously [25]. All His-tagged shFcγRs were purified using Ni-nitrilotriacetic acid affinity chromatography, and their purities and molecular weights were confirmed using SDS–PAGE.

### Antibody binding to shFcγRs

The binding activity of IgG4 mutants toward a series of soluble FcγRs was measured using enzyme-linked immunosorbent assay (ELISA). ELISA plates were coated with 5 μg/mL of anti-tetra-His antibodies (Qiagen) in a carbonate–bicarbonate buffer (Sigma). After blocking with Super Block (Thermo Scientific), purified receptors were added, and the plates were incubated overnight at 4°C. Subsequently, the wells were washed with PBS containing 0.05% Tween 20 (wash buffer), and then serial dilutions of IgGs in 10% Block Ace were added and incubated at room temperature for 2 h. Lastly, after washing with the wash buffer, bound IgGs were detected using peroxidase-labeled goat anti-human kappa antibodies (Southern Biotech) with TMB+ (Dako) as the substrate. The reaction was stopped by adding 0.5 M sulfuric acid (Wako), and the absorbance at 450 nm was measured on an ARVO plate reader (Perkin Elmer).

### CDC assay

CDC assay was performed as described previously [26]. The target cells (5 × 10^4^) were briefly incubated with various concentrations of anti-CD20 antibody and human serum (Sigma), included as the source of complement, in supplemented RPMI-1640 medium for 2 h at 37°C in 96-well flat-bottomed plates. Next, the cell-proliferation reagent CellTiter-Glo (Promega) was added (15 μL/well), and the plates were further incubated for 2 h and then used for detecting live cells. The absorbance (A450–A650) was measured using an ARVO plate reader, and cytotoxicity was calculated according to this formula:

$$\%cytotoxicity=100\times \frac{E-S}{M-S},$$

where E is the absorbance of the experimental well; S is the absorbance in the absence of the monoclonal antibody, i.e., for cells incubated with medium and complement alone; and M is the absorbance of the medium and complement in the absence of target cells and antibody.

### Fab-arm exchange *in vitro*

Bispecific antibodies composed of kappa/lambda light chains were measured using ELISA. Anti-CD20 IgG1, IgG4, IgG4E_K370E, IgG4E_R409K, IgG4PE, IgG4PE_K370E, or IgG4PE_R409K, and anti-CD20 IgG4 containing the lambda light chain were mixed at a 1:1 molar ratio and incubated with 1 mM reduced glutathione at 37°C for 12 h. The final concentration of each antibody was 50 μg/mL, and the mixed antibodies were diluted with ice-cold PBS. ELISA plates were coated with goat anti-human kappa antibodies (Southern Biotech) in a carbonate–bicarbonate buffer (Sigma). After blocking with Super Block (Thermo Scientific), serial dilutions of IgGs in 10% Block Ace were added to the wells, and the plates were incubated at room temperature for 1 h. The wells were washed with PBS wash buffer (see previous section on “Antibody binding to shFcγRs”), and then bispecific antibodies composed of kappa/lambda light chains were detected using peroxidase-labeled goat F(abʹ)2 anti-human lambda antibodies (Southern Biotech) with TMB+ as the substrate. The reaction was stopped by adding 0.5 M sulfuric acid, and the 450-nm absorbance was measured on an ARVO plate reader.

### Fab-arm exchange *in vivo*

All animal procedures were performed in accordance with the protocols approved by the Institutional Animal Care and Use Committee of Kyowa Hakko Kirin Co., Ltd. (Approval Number: A-126). We used 6-week-old female Balb/c nude mice (CHARLES RIVER LABORATORIES JAPAN, INC.). The mice were housed at 19–25°C and 30%–70% humidity under a 12/12-h light/dark cycle and were provided *ad libitum* access to tap water and food before the experiments. Anti-VLA4 IgG1, IgG4, IgG4PE, or IgG4PE_R409K containing the kappa chain, and anti-CD20 IgG4 containing the lambda light chain were intravenously administered at a dose of 100 μg/mouse each. Ten days after antibody administration, approximately 70 μL blood was collected from each mouse by orbital sampling. The mice were euthanized by cervical dislocation, and all efforts were made to minimize animal suffering. Blood samples were used for the detection of kappa/lambda bispecific antibodies, as described above. Whole IgGs were measured using polyclonal rabbit anti-human IgG (Dako A0423) and polyclonal rabbit anti-human IgG/HRP (Dako P0214), and sandwich ELISA was performed.

### Generation of anti-CD20 and anti-VLA4 antibody variants

Expression vectors for anti-CD20 IgG rituximab variants containing lambda light chains and anti-VLA4 IgG natalizumab (US patent 5,840,299) variants were constructed in the expression vector N5KG (Biogen IDEC). Antibodies were expressed and purified as described above (see “Construction, expression, and purification of antibodies” section). To quantify bispecific antibodies, an anti-CD20 kappa/lambda antibody was generated. The expression vectors of anti-CD20 kappa and lambda antibodies were co-transfected into CHO-S cells (Invitrogen), and the bispecific anti-CD20 kappa/lambda antibody was purified from the supernatants using Protein A-conjugated Sepharose and strong cation-exchange columns (GE Healthcare).

## Results

### Identification of an amino acid critical for aggregation at low pH in human IgG4

**Fig 1:**
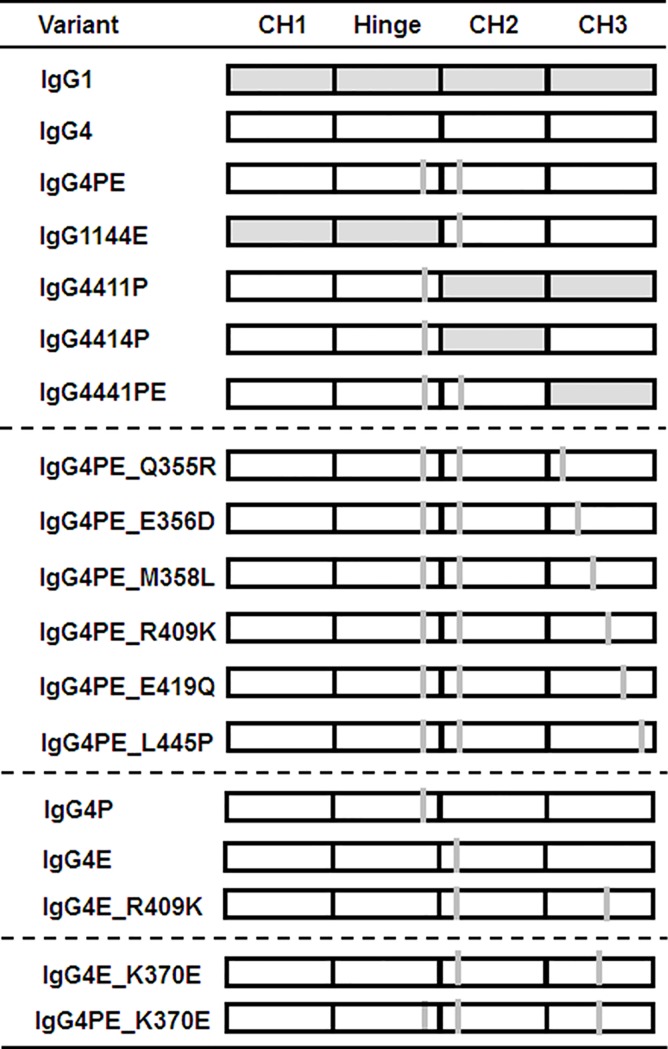
Schematic of domain subclass-switched/swapped and/or substituted derivative antibodies constructed in this study. Shaded and hollow rectangles represent domains derived from human IgG1 and IgG4, respectively, whereas gray bars depict amino acid substitutions. All antibodies shared variable regions of anti-CD20 antibody and light-chain constant region of the kappa isotype.

**Fig 2:**
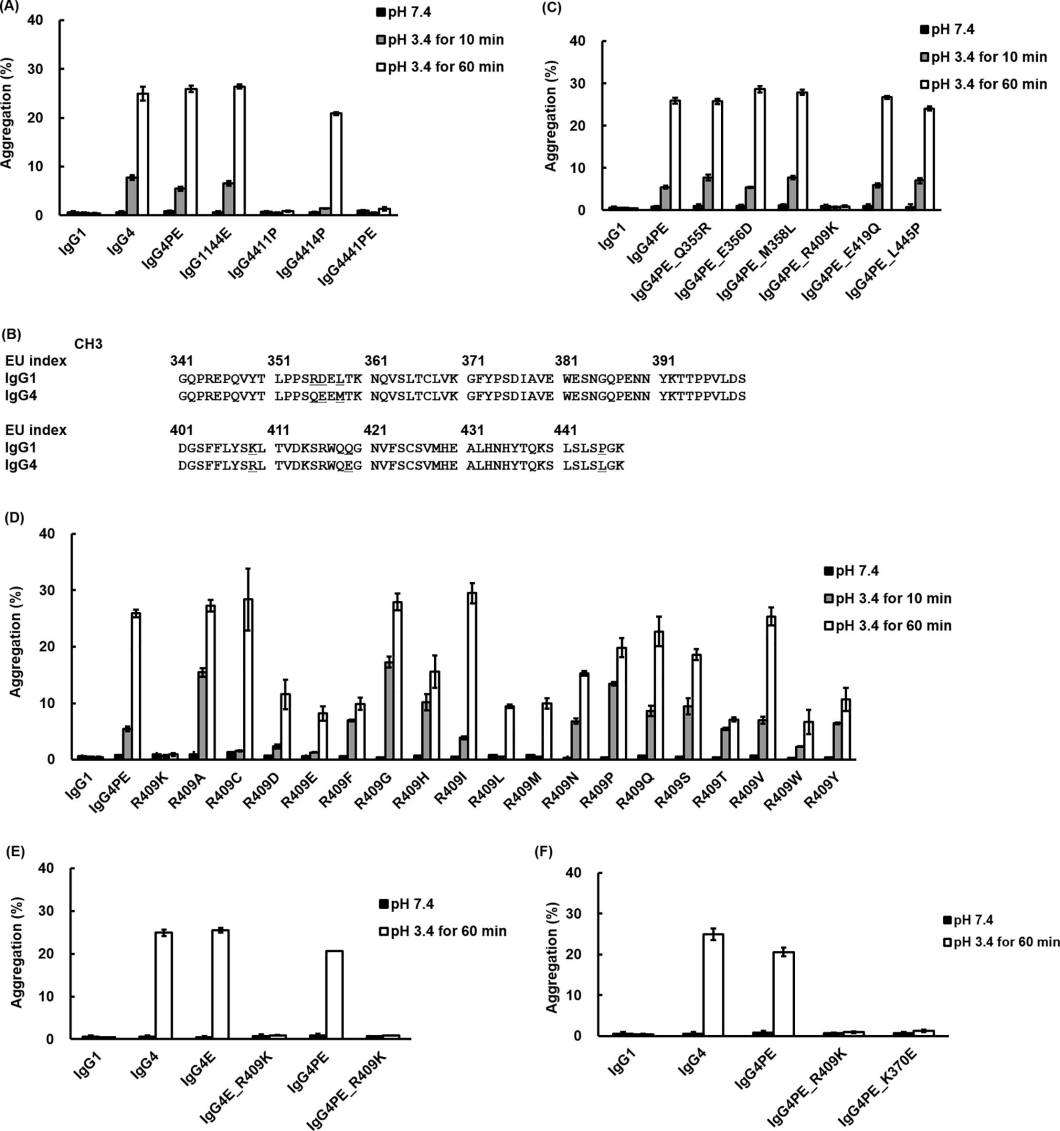
Identification of an amino acid responsible for low-pH-induced aggregation in human IgG4. (A) Identification of the domain critical for aggregate formation through domain swapping between IgG1 and IgG4. SEC analysis of the percentage of aggregation following incubation at pH 3.4 for 10 and 60 min at 37°C. (B) Alignment of the CH3 domains of human IgG1 and IgG4. (C) Identification of the amino acid in CH3 domain that is responsible for acid-induced aggregation. (D) Effect of amino acid substitution at position 409 on acid-induced aggregation. (E) Effect of S228P or L235E mutation on acid-induced aggregation. (F) Effect of K370E mutation on acid-induced aggregation. Data are presented as means ± SD of experiments performed in triplicate. Black bars: initial data; gray and white bars: pH 3.4 for 10 and 60 min at 37°C, respectively.

To identify the domain responsible for aggregation in human IgG4, we performed domain-subclass conversion of the CH regions (Fig 1). Antibodies containing hybrid constant regions, IgG1144E, IgG4411P, IgG4414P, and IgG4441PE, were generated by combining human IgG1 and IgG4PE, and aggregate formation was then investigated using SEC after incubation at pH 3.4 for 10 and 60 min at 37°C. Representative SEC-HPLC chromatograms of IgG4PE are shown in S1 Fig. The control samples of all IgGs were of high quality with less than 1.5% aggregation (Fig 2A). Whereas IgG1 exhibited high physical stability, IgG4 and IgG4PE showed considerable aggregation. The results agree with that of nivolumab harboring the S228P mutation in IgG4 and producing an aggregate content of 30% at pH 3.5 [23]. The IgG1144E and IgG4414P variants also exhibited insufficient reduction of aggregation at low pH. Notably, in IgG4411P and IgG4441PE, which contained the CH3 domain of IgG1, the aggregation resistance of IgG4 was markedly enhanced and was comparable to that of IgG1. These results suggested that aggregate formation in IgG4 involves the CH3 domain.

To identify the aggregation-prone amino acid in IgG4, we focused onsix6 amino acid residues (at Kabat positions 355, 356, 358, 409, 419, and 445) that differ in IgG1 and IgG4 in the terms of CH3 domain (Fig 2B) and constructed derivatives harboring substitutions in the CH3 domain (Fig 1). In IgG4PE_R409K, aggregate formation at low pH was effectively prevented and occurred in the same extent as that of IgG1. However, several other mutations in IgG4PE at Q355R, E356D, M358L, E419Q, and L445P did not prevent the aggregation (Fig 2C). We also found that acid exposure had no effect on the degradation of these antibodies (S2 Fig). Moreover, this inhibitory effect on aggregate formation was confirmed in our investigations of IgG4PE_R409K that featured either a different variable region (anti-VLA4 antibody; S3 Fig) or lambda light chains (S4 Fig). We further examined how substitutions of other amino acids at position 409 in IgG4PE affect acid-induced aggregation, revealing that no substitution prevented acid-induced aggregate formation as effectively as R409K. However, in certain substitutions, such as R409E, R409F, R409L, R409M, R409T, R409W, and R409Y, partial inhibition of acid-induced aggregation was observed for 60 min at pH 3.4 and 37°C (Fig 2D).

We determined whether the R409K mutation alone prevented acid-induced aggregation of IgG4. IgG4E_R409K, which lacks the S228P mutation, was as resistant to aggregation as the variant harboring the combination of S228P and R409K. Meanwhile, the combination of the R409K mutation and L235E, which reduces FcR binding, inhibited acid-induced aggregation to the same extent as that in IgG4PE_R409K (Fig 2E). Our results suggested that the CH3 domain is involved in the aggregate formation of IgG4, and we thus examined whether aggregation was also inhibited by the K370E mutation, which was reported as a stabilizing mutation in CH3 that suppresses aggregate formation [14]. In IgG4PE_K370E, acid-induced aggregation was prevented following incubation at pH 3.4 for 60 min at 37°C (Fig 2F).

### Thermodynamic stability

**Fig 3:**
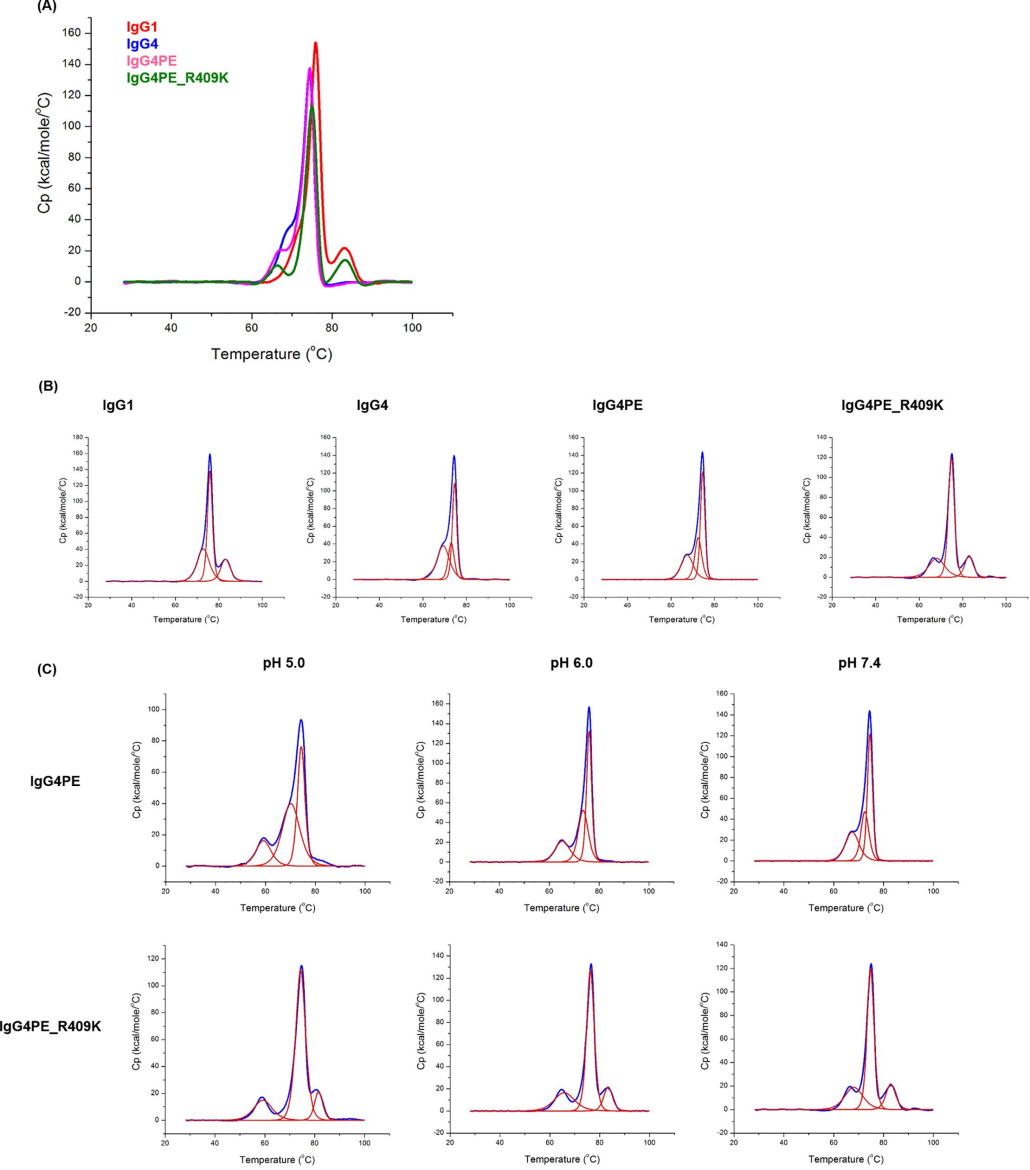
DSC analysis of thermodynamic stability. (A) DSC charts of anti-CD20 IgG1, IgG4, IgG4PE, and IgG4PE R409K antibodies at pH 7.4 with PBS buffer. (B) Post-deconvoluted DSC charts of IgG1, IgG4, IgG4PE, and IgG4PE R409K antibodies at pH 7.4 with PBS buffer. (C) Post-deconvoluted DSC charts of IgG4PE and IgG4PE R409K antibodies at different pH conditions including 5.0, 6.0, and 7.4.

To determine whether the R409K mutation in IgG4 affects thermodynamic stability, we obtained the DSC thermograms of four distinct IgGs, namely IgG1, IgG4, IgG4PE, and IgG4PE_R409K in PBS buffer of pH 7.4 (Fig 3A). To identify the peaks corresponding to specific antibody regions, we obtained DSC deconvolution thermograms (Fig 3B). Table 1 shows the thermal unfolding midpoint (*T*m) values. For IgG1, the *T*m values of CH2, Fab, and CH3 were approximately 72.9, 75.9, and 83.1°C, respectively. For IgG4, IgG4PE, and IgG4PE_R409K, the *T*m values of CH2 shifted to a lower temperature than that of IgG1, and the *T*m values of Fab in the IgG4 variants shifted to a slightly lower temperature than that of IgG1. Conversely, the melting transition of CH3 in IgG4PE_R409K shifted to a higher temperature, approximately 83°C (as in IgG1), than those of IgG4 and IgG4PE, owing to the R409K mutation within the CH3 domain. To investigate the mechanism of the R409K mutant stability in low pH, we also performed DSC analyses of IgG4PE and IgG4PE R409K at pH 5.0 and 6.0 (Fig 3C). Table 2 shows the *T*m values. The *T*m values of CH3 in IgG4PE R409K was higher than that of IgG4PE at pH 5.0 (81.6°C vs. 70.3°C) and pH 6.0 (83.4°C vs. 73.6°C). For IgG4PE and IgG4PE_R409K, the *T*m values of CH2 at pH 5.0 shifted to a lower temperature than those of at pH 7.4. These data suggest that the R409K mutation increases the *T*m values of CH3 in low pH, thereby enhancing resistance to low pH stress.

**Table 1:** DSC measurements of the melting transition (Tm) of IgG variants at pH 7.4.

	Tm (°C)		
Domain	CH2	Fab	CH3
IgG1	72.9	75.9	83.1
IgG4	69.4	74.6	73.0
IgG4PE	67.4	74.5	72.5
IgG4PE R409K	68.1	74.8	82.9

**Table 2:** DSC measurements of the melting transition (Tm) of IgG4PE and IgG4PE R409K at pH 5.0, 6.0, and 7.4.

	Tm (°C)								
	CH2			Fab			CH3		
pH	5.0	6.0	7.4	5.0	6.0	7.4	5.0	6.0	7.4
IgG4PE	59.2	65.3	67.4	74.4	76.0	74.5	70.3	73.6	72.5
IgG4PE R409K	59.3	65.8	68.1	74.3	76.3	74.8	81.6	83.4	82.9

### Fab-arm exchange *in vitro* and *in vivo*

**Fig 4:**
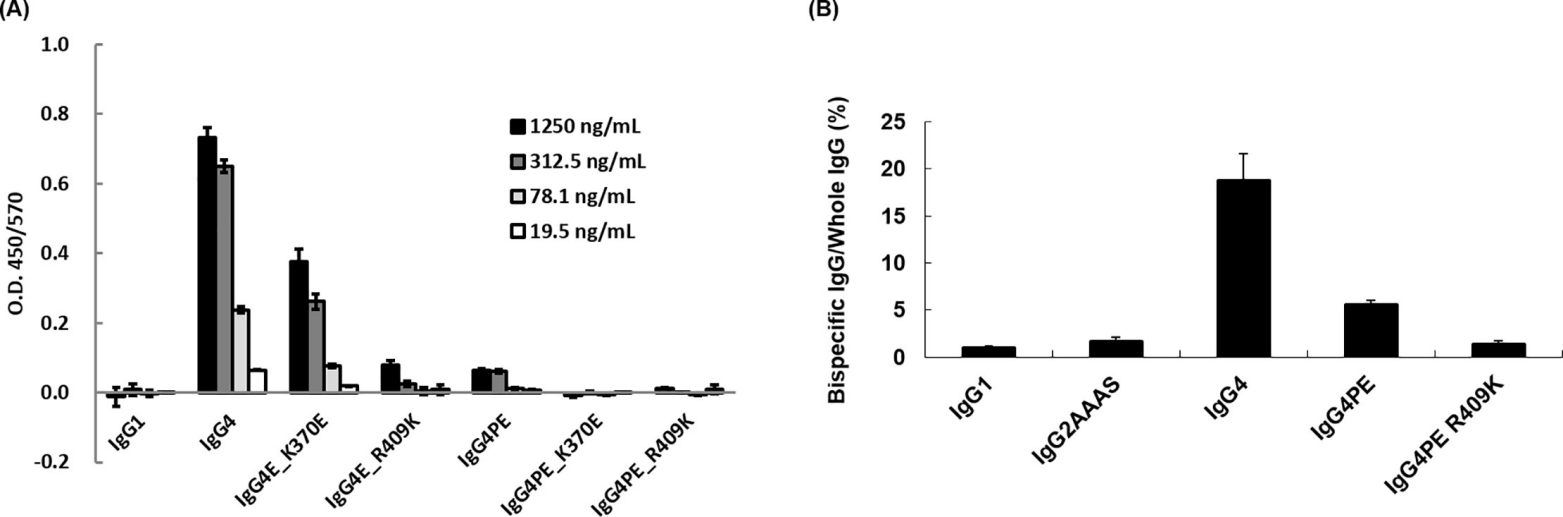
Effect of R409K mutation on Fab-arm exchange *in vitro* and *in vitro*. (A) Effect of R409K mutation on Fab-arm exchange *in vitro*. Bispecific anti-CD20 antibodies composed of kappa/lambda light chains were detected using ELISA. Mixtures of anti-CD20 IgG1, IgG4, IgG4PE, IgG4PE_K370E, or IgG4PE_R409K containing kappa light chain and anti-CD20 IgG4 containing lambda light chain were incubated with 1 mM reduced glutathione at 37°C for 12 h. Goat anti-human kappa antibodies were coated on 96-well immunoplates, which were then incubated with diluted mixtures of antibodies; the bispecific antibody was detected using peroxidase-labeled goat F(abʹ)2 anti-human lambda antibodies. Results are shown as means ± SD of experiments performed in triplicate. (B) Effect of R409K mutation on Fab-arm exchange *in vivo*. Nude mice (n = 5) were injected with a mixture of anti-VLA4 IgG1, IgG2AAAS, IgG4, IgG4PE, or IgG4PE_R409K containing kappa light chain and anti-CD20 IgG4 containing lambda light chain. IgG2AAAS: IgG2 variant harboring V234A/G237A/P331S substitutions. Blood samples were collected at 10 days after antibody administration and were used for the detection of kappa/lambda bispecific antibodies or whole IgG. Data bar and error bar: average and standard error, respectively.

We examined how Fab-arm exchange was affected in R409K mutants *in vitro*: Fab-arm-exchanged IgG4 antibodies featuring both kappa and lambda light chains were detected, but in IgG1, Fab-arm exchange was suppressed (Fig 4A). Moreover, in IgG4, the hinge-stabilizing S228P mutation suppressed Fab-arm exchange. Analysis of the influence of CH3-stabilizing mutations on Fab-arm exchange revealed that whereas the K370E mutation only partially suppressed Fab-arm exchange, R409K drastically inhibited the exchange. Furthermore, in the variants with combined hinge-stabilizing and CH3-stabilizing mutations, IgG4PE_K370E and IgG4PE_R409K, Fab-arm exchange are markedly suppressed.

We next examined *in vivo* the effect of combination of hinge stabilization and CH3 stabilization (Fig 4B). As with the *in vitro* results, the IgG4 antibodies were found to harbor both kappa and lambda light chains, but Fab-arm exchange was again suppressed in IgG1 antibodies. Moreover, in IgG4PE, Fab-arm exchange was strongly suppressed, and our results suggested that the R409K substitution in IgG4PE exerts an additive inhibitory effect.

### Antigen (FcγR)-binding profiles and CDC activity

**Fig 5:**
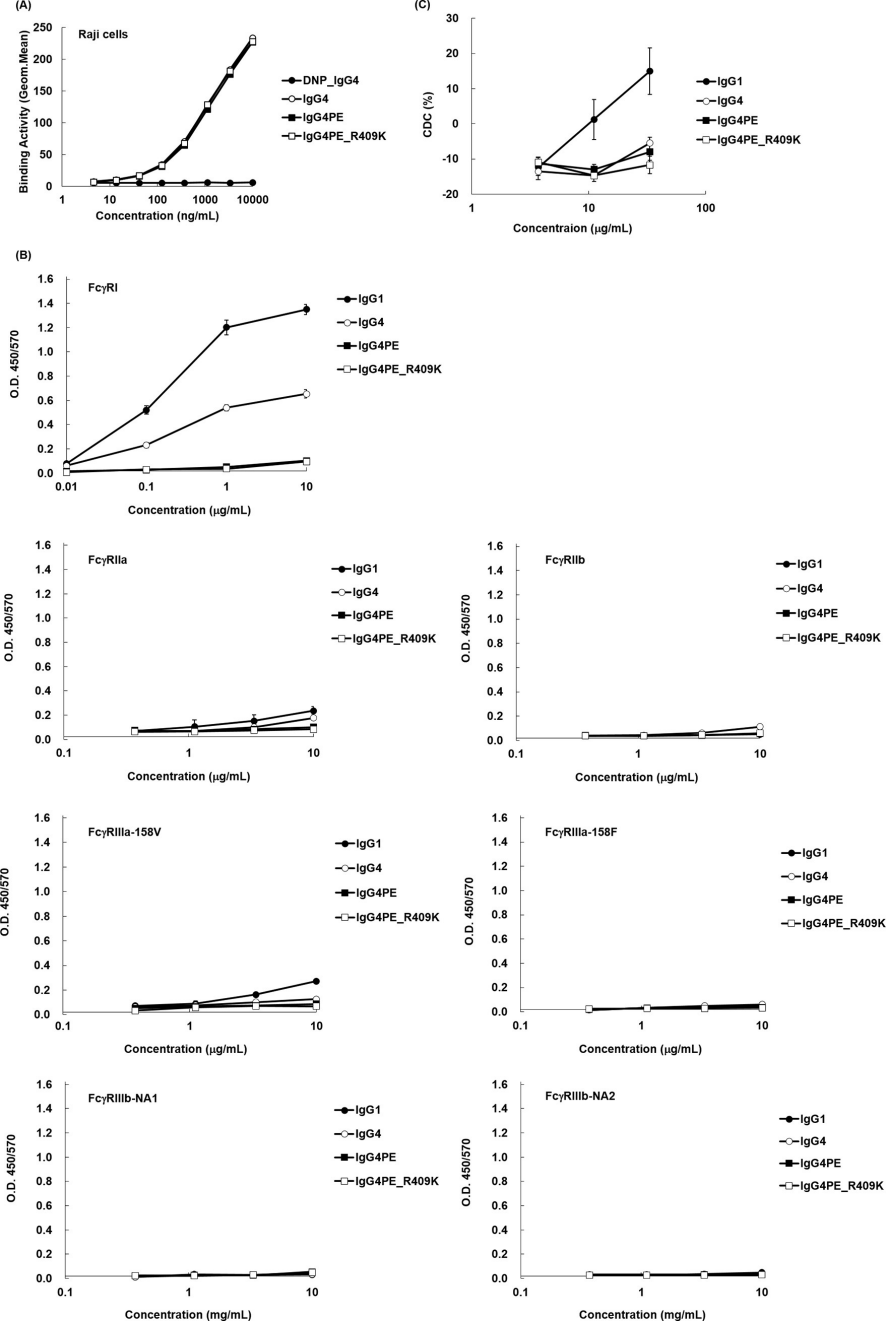
Effect of R409K mutation on antigen (FcγR) binding and CDC activity. (A) Binding of IgG4 mutants to Raji cells, measured using flow cytometry; cells were stained with IgG4 (open circles), IgG4PE (closed squares), IgG4PE_R409K (open squares), or isotype control IgG (closed circles), and the binding was detected using an Alexa Fluor 488-labeled secondary antibody. (B) ELISA for measuring the binding activity of IgG4 mutants toward human FcRs. Each FcγR was coated on 96-well immunoplates by using anti-tetra-His antibodies, and the plates were incubated with the indicated concentrations of IgG1 (closed circles), IgG4 (open circles), IgG4PE (closed squares), or IgG4PE_R409K (open squares); binding was detected using a peroxidase-labeled secondary antibody. (C) CDC of IgG4 mutants against Raji cells. After 2-h incubation, cytotoxicity was measured using CellTiter-Glo. Means ± SD of triplicates are shown.

To determine whether R409K mutation in IgG4 affects the biological functions of the antibody, we investigated antigen-binding profiles. Flow cytometric analysis performed using CD20-positive Raji cells revealed that IgG4, IgG4PE, and IgG4PE_R409K showed similar CD20-binding activity (Fig 5A). We next used ELISA to measure the binding of the anti-CD20 IgG variants to shFcγRI, shFcγRIIa, shFcγRIIb, shFcγRIIIa-158V, shFcγRIIIa-158F, shFcγRIIIb NA1, and shFcγRIIIb NA2. In agreement with previous findings [6], wild-type IgG4 showed lower binding affinity for shFcγRI than did IgG1, and a large loss of affinity was observed in the case of IgG4PE, which exhibited low binding affinity for all FcγRs; the FcγR-binding profiles indicated similar antigen binding by IgG4PE and IgG4PE_R409K (Fig 5B). To examine whether the R409K mutation affects CDC activity, we assessed this activity by using human serum as the complement source (Fig 5C), revealing that the CDC activity of IgG4 was lower than that of IgG1, which is in agreement with previous findings [6]. Moreover, similar to wild-type IgG4 and IgG4PE, IgG4PE_R409K exhibited low CDC activity (Fig 5C).

## Discussion

Human IgG4 antibodies are suitable for use in the potential low-effector-function format of therapeutic antibodies. However, IgG4 antibodies are susceptible to aggregation under low-pH conditions. The aggregation of therapeutic antibodies influences both the efficacy of the therapeutic treatment and safety, including immunogenicity, infusion reaction, and complement activation. Because therapeutic antibodies are exposed to low pH during the purification step in their manufacturing process, one of the key areas of research in this field is focused on the suppression of low-pH-induced antibody-aggregate formation. In this study, we developed an engineered IgG4 antibody that exhibits reduced aggregation under low-pH conditions.

**Fig 6:**
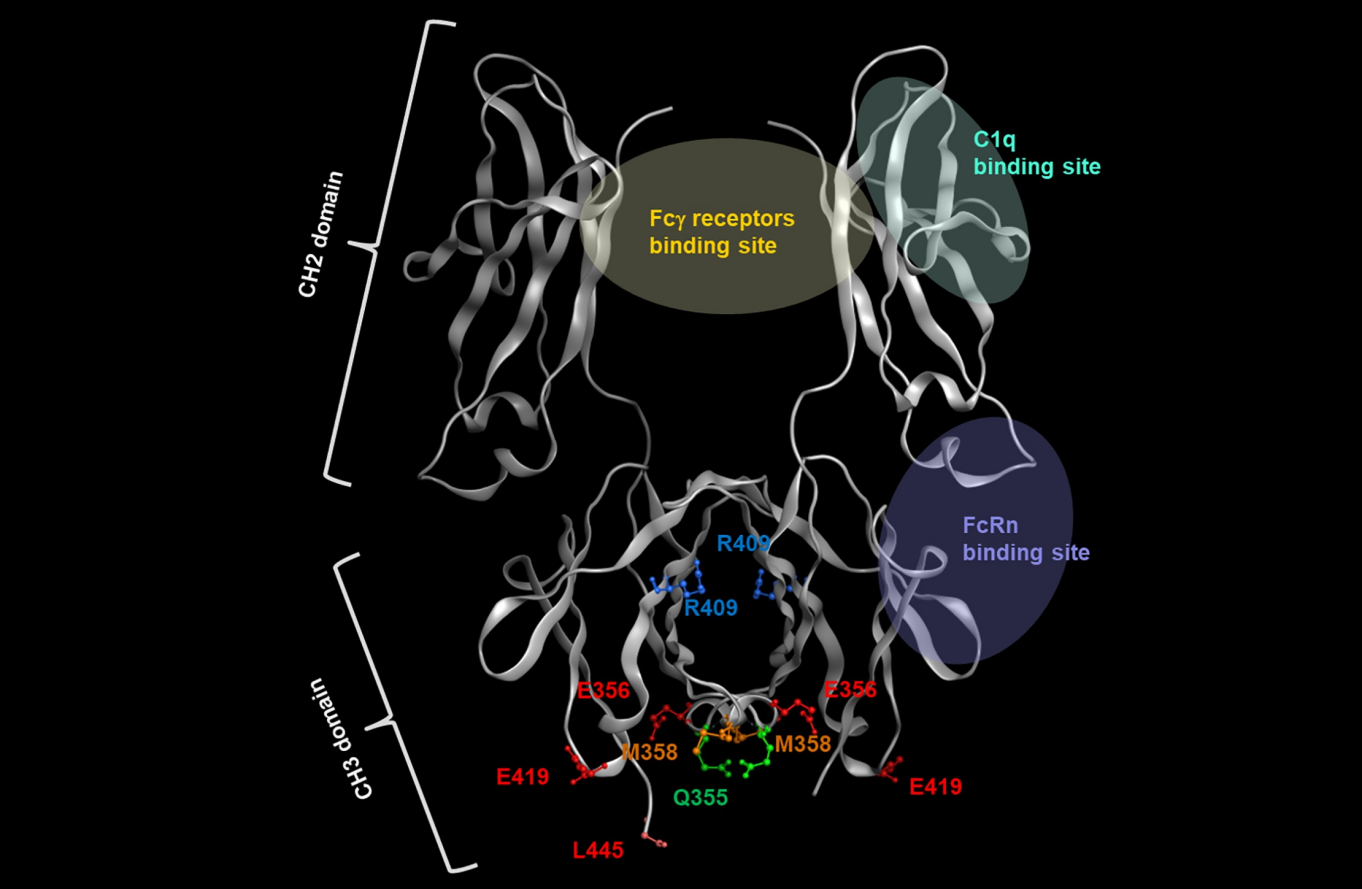
Structure of the Fc region of human IgG4. The model was built using published structural data of human IgG4 (Protein Data Bank accession code: 4C54). Amino acids of human IgG4 described in the Discussion are highlighted in a different color.

We found that the domains or amino acids related to aggregate formation could be identified by constructing chimeric antibodies composed of constant regions of IgG1 and IgG4: swapping in of IgG1 regions into IgG4 resulted in suppression of low-pH-induced aggregation of IgG4. This inhibition of aggregation was confirmed in IgG4441PE, in which the IgG4 CH3 was changed to the CH3 from IgG1; this suggested that aggregate formation in IgG4 is caused by the CH3 domain. The results agree with a report that CH3 plays the most critical role in aggregation under acidic conditions [27]. Moreover, the hydrophobic patch in IgGs is one of the causes of aggregate formation and the patch can be predicted using an *in silico* approach [28, 29], and the hydrophobic patch, which includes Ile253 in the CH2 domain in IgG4, has been reported to be more hydrophobic on the side patch as compared with the corresponding region in IgG1 and IgG2 [30]. However, in our IgG4414P variant here, in which the CH2 domain of IgG4 was replaced with that of IgG1, no notable inhibitory effect on acid-induced aggregate formation was observed. Therefore, aggregate formation appears to be promoted by a structural change of the CH3 domain under acidic conditions. Our results further suggested that amino acid residue 409 of IgG4 is involved in the interaction between CH3 domains and is related to aggregate formation, because among the 6 amino acid residues (355, 356, 358, 409, 419, and 445) that differ in the CH3-domain sequence of IgG1 and IgG4, the Fc-Fc interaction surface includes only position 409 (Fig 6), according to structural analysis [29]. Thus, as expected, conversion of Arg409 of IgG4 to a Lys as in IgG1 caused a suppression of aggregate formation. The other 5 amino acids are located on the molecular surface and are not contained in the aggregation motif [28], which is consistent with our results. Davies et al. analyzed the crystal structure of the Fc from IgG1 and IgG4, and showed that in IgG4, the Arg409 guanidinium group oriented toward Asp399ʹ of the other heavy chain, with which it engages in an electrostatic interaction, but this Arg409-Asp399ʹ distance is greater than the Lys409-Asp399ʹ distance in IgG1; Davies et al. also showed that Arg409 disrupts the network of hydrogen bonding through the water at Asp399ʹ, Lys370ʹ, and Asn390, which results in the Fc-Fc packing in IgG4 CH3 being weaker than that in IgG1 CH3 [15, 31]. Therefore, the R409K mutation could suppress aggregate formation through the strengthening of Fc-Fc packing followed by a reduction of the exposed hydrophobic area of the Fc domain at low pH.

As a further evaluation of antibody stability, we performed DSC analysis to measure thermal stability. It revealed enhanced stability of the CH3 domain in the R409K mutant at pH 5.0, 6.0, and 7.4, further supporting our results. Moreover, the CH3 of IgG4PE has been reported to be less thermostable than that of IgG1 [22], which is consistent with our results. The CH2 domain was found to be the least stable among all IgG domains [22] and might be involved in aggregate formation at low pH. However, our results here showed that the *T*m values of CH2 in IgG4PE and IgG4PE R409K were similar to those of at pH 5.0. We also confirmed that in IgG4414P, wherein IgG4 CH2 was changed to CH2 of IgG1, aggregate formation was not notably suppressed. The low conformational stability and the structural change are initiated in CH2 of IgG4. However, aggregate formation is suppressed by the conformational stability of CH3 in the R409K mutant, which may have led to structural instability, and eventually to irreversible structural changes, initiating aggregate formation. Aggregation of monoclonal antibodies depends on their conformational and colloidal stabilities [32]; Yageta et.al. reported the conformational and colloidal stabilities of isolated antibody constant domains under a wide range of pH and salt conditions. However, a high concentration at 50 μM and pH of 2 or 3 affected the colloidal stability of CH2 and CH3 [27]. We measured low-pH-induced aggregation at an antibody concentration of 0.2 mg/mL. The conformational stability could be pre-dominant for aggregation, and the effect of colloid stability appears to be small in this study. However, we recommend including accelerated tests to evaluate how the R409K mutant affects the conformational and colloidal stabilities under a wide range of pH and salt conditions at high antibody concentrations.

Certain substitutions, such as R409E, R409F, R409L, R409M, R409T, R409W, and R409Y, were also found to potentially inhibit acid-induced aggregation, although not completely. According to Rose et al., in an *in silico* mutagenesis analysis of the energetics of the CH3–CH3 interface, all amino acids except Asp were favorable residues at position 409 in the dimer with the scoring value tending to be high for Met or Trp [33]. Our results suggested that the inhibitory effect on aggregate formation in the R409M and R409W variants was produced by the hydrophobic strengthening of the CH3–CH3 interaction. Rose et al. elaborated that the R409K variant exhibited maximal dimerization among all the variants tested, which is also consistent with our results, and that the mutations introduced at position 370 in IgG4 led to an increase in dimerization strength [33]. K370E mutation was also found in our study to be potent at preventing acid-induced aggregation. These findings strongly suggest that the CH3–CH3 interaction is critical for inhibition of low-pH-induced aggregate formation.

Our results are also consistent with previous reports indicating that pH-dependent stability can vary according to the variable region but not according to the IgG subclass [34]. Our findings provide key insights into the Fc-Fc packing that occurs in the CH3 domain during acid-induced aggregation specifically of IgG4. Intriguingly, a Lys is present at position 409 in IgG2, but IgG2 is prone to aggregation under acidic conditions. The amino acids responsible for aggregate formation might be different for each subclass. The IgG2 structure presents highly distinct dynamic CH2–CH3 interfaces as compared with the IgG1 structure [35]. Whereas acid-induced IgG2 aggregation was partially reversed during neutralization, IgG4 aggregation was irreversible [30]. Thus, although Lys is present at position 409 in IgG2, it might be specifically involved in IgG4 stabilization following the R409K substitution, and the mechanism of low-pH-induced aggregate formation might differ among the IgG subclasses. Furthermore, a Lys is present at position 409 in IgG3, but IgG3 is still prone to aggregation under acidic conditions. A recent study has reported that two amino acid mutations in the CH3 domain, N392K and M397V, decreased aggregation during low pH conditions [36]. However, our study only analyzed IgG4 aggregation under low-pH treatment. Because aggregation can also occur during storage, long-term stability must be investigated to elucidate the effectiveness of the R409K variant as a potential therapeutic antibody.

We found that Fab-arm exchange was additively prevented by a combination of hinge stabilization of IgG4 and enhancement of CH3 interaction. Our findings suggested that either S228P or R409K mutation alone, or a combination of S228P and R409K prevented Fab-arm exchange. However, K370E alone produced a partial inhibitory effect, and additive suppression was obtained with the combination of S228P and K370E mutations. *in vitro* assay conditions such as the reduced concentration of glutathione used might not precisely reflect the *in vivo* environment, and the proportion of Fab-arm exchange can vary depending on the concentration [10, 37, 38]. Thus, further investigation of this effect is necessary.

Antibody engineering can potentially affect the biological properties that underpin the efficacy and safety of a therapeutic antibody being developed. However, our study revealed that the R409K substitution exerted no effect on the biological properties of IgG4, at least on antigen binding and effector function. Here, the FcγR-binding pattern was unaffected by the R409K mutation. The CH2 domain is a major FcγR-binding site [39–42], suggesting that R409K, an amino acid modification in CH3, does not influence structural changes related to the FcR-binding site. Human IgG4 exhibits low effector functions because of its binding to low-affinity FcγR, and IgG4 binds to FcγRI as effectively as IgG1 and IgG3 [43]. However, this FcγR-binding ability can be diminished through L235E and G237A substitutions in IgG4 [44, 45]. Enhancement of the Fc–Fc interaction by R409K mutation did not alter the influence of L235E modification on low-affinity FcR binding, and thus additional FcγR-related effects of the R409K mutation appeared minimal. However, we only analyzed affinities based on ELISA results, and we recommend further analysis of binding affinity and FcγR-related effector functions to comprehensively elucidate the potential of the R409K mutant as a therapeutic antibody.

We found that CDC activity was not markedly affected in R409K mutants. IgG4 was reported to exhibit considerably diminished complement activation because of its low affinity for C1q. In human IgG, the residues in CH2 domain that are responsible for forming the C1q-binding site are L235, D265, D270, K322, P329, and P331 [42, 46–50]. This is distinct from the binding site in the R409K variants. However, recent studies reported that domain swapping of IgG1 and IgG3, which converted the Val at position 397 of IgG1 to Met as that of IgG3, enhanced CDC activity [26]. Its underlying mechanism might involve the weakening of CH3 interaction and enhancement of CH3 flexibility [38]. In contrast, the R409K mutation in IgG4 strengthens CH3 interaction and eliminates its flexibility, which is consistent with the result that the mutation does not affect the CDC activity of the antibodies.

Currently, an engineered IgG scaffold lacking the effector function is recognized as a suitable format for use as antagonist or agonist antibodies [6], and it is critical that the development of such antibodies considers stability. Recent studies have reported stable IgG1-based variants featuring a scaffold lacking effector functions including N297G or L234A, L235A, and P329G [51, 52]. An IgG1 antibody exhibiting low effector function is one candidate format. The agonist or antagonist activity of an antibody might differ depending on its subclass [53–55], but a subclass suitable for the required function of the antibody must be selected. Furthermore, studies on factors affecting IgG1 stability have been reported, such as those describing an increase of degradation products under low-pH conditions and radical-induced hinge cleavage; hence, further investigation of these factors is necessary [22, 56–60]. To date, no therapeutic IgG4 antibody harboring the R409K mutation has been approved for clinical use. However, one of the human IgG4 allotypes contains a Lys residue at position 409 [61], and this has not yet been reported to cause any disease, including any IgG4-related disease in humans [62]. This suggests that the R409K mutant is potentially safe for use in the therapeutic-antibody format, but further nonclinical studies are necessary to completely elucidate the therapeutic potential of the R409K mutant.

In summary, we demonstrated through protein engineering the biological importance of R409K mutation in the resistance of human IgG4 to acid-induced aggregation. We showed that the biophysical profiles of IgG4PE_R409K, including binding to antigens (FcγRs) and CDC activity, were similar to those of IgG4PE. Therefore, the R409K variant has potential for use in the low-effector-function format of therapeutic antibodies. Our findings provided new information on antibody stabilization and offered new insights for the design and engineering of therapeutic antibodies with enhanced stability.

## Supporting information

S1 Fig HPLC-SEC profile of IgG4PE. (A) SEC profile of IgG4PE at pH 7.4. (B) SEC profile of IgG4PE following incubation for 60 min at pH 3.4 and 37°C.
(TIF)

S2 Fig Effect on acid-induced degradation in anti-CD20 IgG rituximab variants. Acid-induced degradation of rituximab variants was analyzed using SEC. Antibodies were treated with a 0.1 M citric acid buffer (pH 2.7) and adjusted to pH 3.4, incubated at 37°C for 60 min, and then neutralized by adding 500 mM phosphate buffer (pH 8.0). The amounts of the small soluble aggregates formed were analyzed using SEC-HPLC. Data are presented as means ± SD of experiments performed in triplicate. Black bars: initial data; grey and white bars: pH 3.4, for 10 and 60 min at 37°C, respectively.
(TIF)

S3 Fig Effect of R409K mutation on acid-induced aggregation in anti-VLA4 IgG natalizumab variants. Acid-induced aggregation of natalizumab variants was analyzed using SEC. Antibodies were treated with a 0.1 M citric acid buffer (pH 2.7) and adjusted to pH 3.4, incubated at 37°C for 60 min, and then neutralized by adding 500 mM phosphate buffer (pH 8.0). The amounts of the small soluble aggregates formed were analyzed using SEC-HPLC. Data are presented as means ± SD of experiments performed in triplicate. Black bars: initial data; white bars: pH 3.4, 60 min at 37°C.
(TIF)

S4 Fig Effect of R409K mutation on acid-induced aggregation in anti-CD20 IgG rituximab variants containing lambda light chains. Acid-induced aggregation of IgG variants containing lambda light chains were analyzed using SEC. Antibodies were treated with a 0.1 M citric acid buffer (pH 2.7) and adjusted to pH 3.4, incubated at 37°C for 60 min, and then neutralized by adding 500 mM phosphate buffer (pH 8.0). The amounts of small soluble aggregates were analyzed using SEC-HPLC. Data are presented as means ± SD of experiments performed in triplicate. Black bars: initial data; white bars: pH 3.4, 60 min at 37°C.
(TIF)
