# Supplementary figures and images for: R409K mutation prevents acid-induced aggregation of human IgG4
Source: PLoS One. 2020 Mar 17;15(3):e0229027. doi: 10.1371/journal.pone.0229027 (PMC7077836; doi:10.1371/journal.pone.0229027)

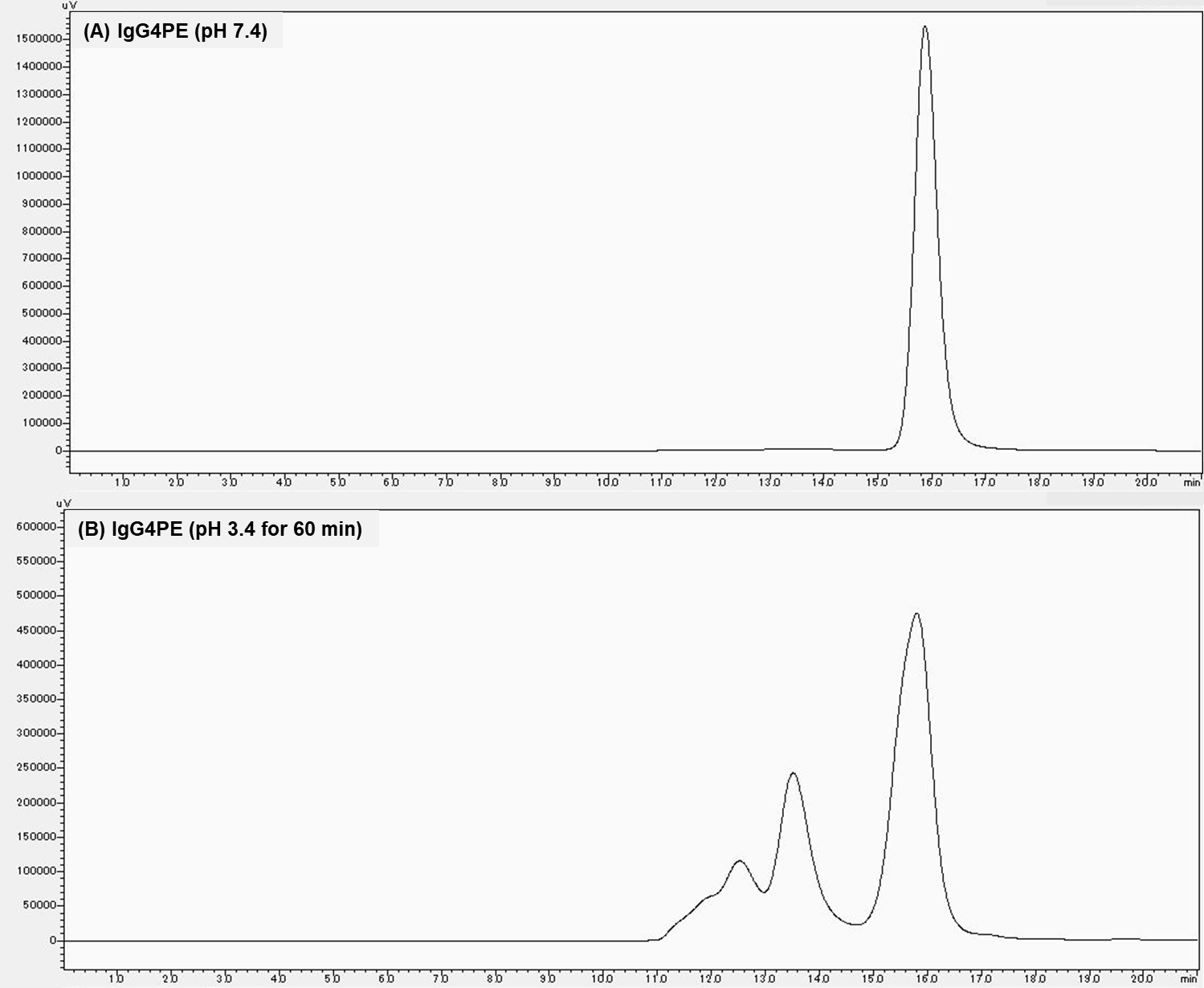

Supplement: S1 Fig — (A) SEC profile of IgG4PE at pH 7.4. (B) SEC profile of IgG4PE following incubation for 60 min at pH 3.4 and 37°C. (TIF) [file pone.0229027.s001.tif]

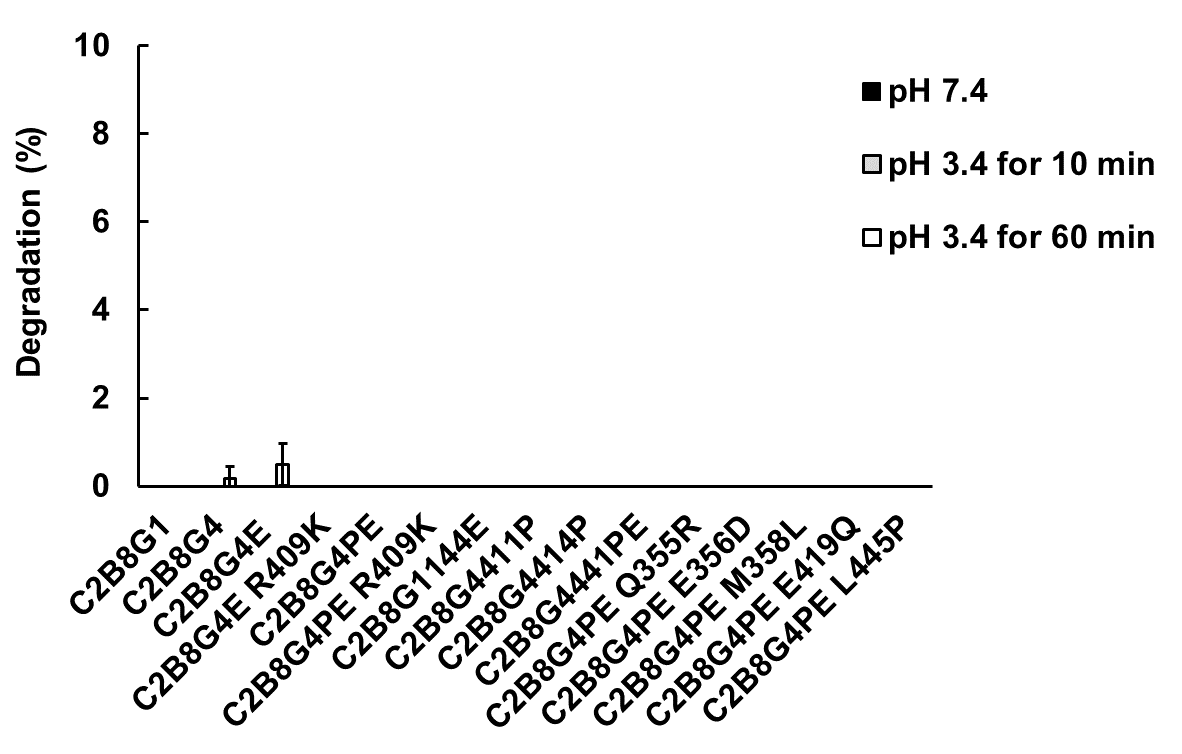

Supplement: S2 Fig — Acid-induced degradation of rituximab variants was analyzed using SEC. Antibodies were treated with a 0.1 M citric acid buffer (pH 2.7) and adjusted to pH 3.4, incubated at 37°C for 60 min, and then neutralized by adding 500 mM phosphate buffer (pH 8.0). The amounts of the small soluble aggregates formed were analyzed using SEC-HPLC. Data are presented as means ± SD of experiments performed in triplicate. Black bars: initial data; grey and white bars: pH 3.4, for 10 and 60 min at 37°C, respectively. (TIF) [file pone.0229027.s002.tif]

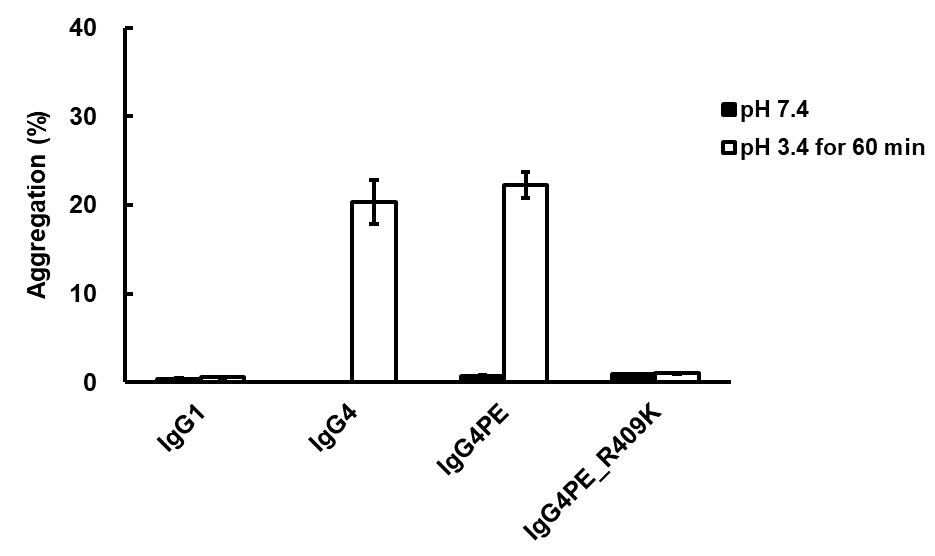

Supplement: S3 Fig — Acid-induced aggregation of natalizumab variants was analyzed using SEC. Antibodies were treated with a 0.1 M citric acid buffer (pH 2.7) and adjusted to pH 3.4, incubated at 37°C for 60 min, and then neutralized by adding 500 mM phosphate buffer (pH 8.0). The amounts of the small soluble aggregates formed were analyzed using SEC-HPLC. Data are presented as means ± SD of experiments performed in triplicate. Black bars: initial data; white bars: pH 3.4, 60 min at 37°C. (TIF) [file pone.0229027.s003.tif]

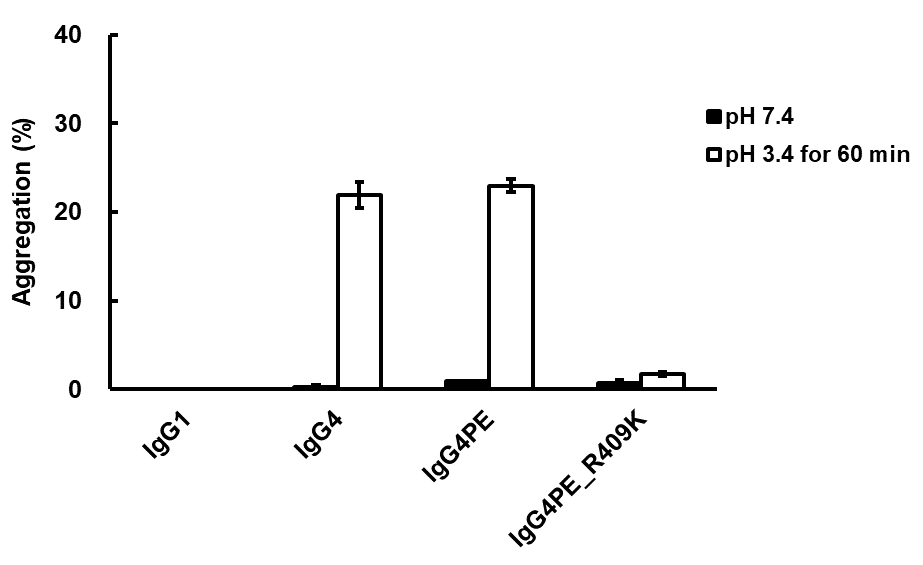

Supplement: S4 Fig — Acid-induced aggregation of IgG variants containing lambda light chains were analyzed using SEC. Antibodies were treated with a 0.1 M citric acid buffer (pH 2.7) and adjusted to pH 3.4, incubated at 37°C for 60 min, and then neutralized by adding 500 mM phosphate buffer (pH 8.0). The amounts of small soluble aggregates were analyzed using SEC-HPLC. Data are presented as means ± SD of experiments performed in triplicate. Black bars: initial data; white bars: pH 3.4, 60 min at 37°C. (TIF) [file pone.0229027.s004.tif]
